# Connexins and cAMP Cross-Talk in Cancer Progression and Metastasis

**DOI:** 10.3390/cancers13010058

**Published:** 2020-12-28

**Authors:** Chang-Xu Chen, Kai-Jun Luo, Jia-Peng Yang, Yun-Chao Huang, Eduardo R. Cardenas, Bruce J. Nicholson, Jean X. Jiang

**Affiliations:** 1 School of Life Sciences, Yunnan University, Kunming 650500, China; changxuchen@mail.ynu.edu.cn; 2 Key Laboratory of the University in Yunnan Province for International Cooperation in Intercellular Communications and Regulations, Yunnan University, Kunming 650500, China; 3 Department of Thoracic Surgery I, The Third Affiliated Hospital of Kunming Medical University/Yunnan Cancer Hospital, Yunnan Cancer Center, Kunming 650500, China; Ian1002@126.com; 4 Joint International Research Laboratory of Regional Tumor in High Altitude Area, Kunming 650118, China; 5 Department of Biochemistry and Structural Biology, University of Texas Health Science Center, San Antonio, TX 78229, USA; CardenasE2@uthscsa.edu (E.R.C.); NicholsonB@uthscsa.edu (B.J.N.)

**Keywords:** connexin, cAMP, cancer, metastasis

## Abstract

**Simple Summary:**

Different connexins play diverse roles in cancers, either tumor-suppressing or tumor-promoting. In lung cancer, Cx43 serves as a tumor suppressor at the early stage, but it can also be a tumor-promotor at an advanced stage and during metastasis. Moreover, other connexins, including Cx26, Cx31.1, and Cx32, can be tumor suppressors. In contrast, Cx30.3 can be a tumor-promotor. The roles of different connexins in different cancers have also been established. Cx43 acts as a tumor suppressor in colorectal cancer, breast cancer, and glioma, whereas Cx32 can be a suppressor in liver tumors and hepatocarcinogenesis. Cx26 can be a tumor suppressor in mammary tumors; in contrast, it can be a tumor-promotor in melanoma. Existing drugs/molecules targeting the cAMP/PKA/connexin axis act to regulate channel opening/closing. Mimic peptides, such as Gap19, Gap26, and Gap 27 block hemichannels, mimetic peptides, and CT9/CT10 and promote hemichannel opening and also hemichannel closing.

**Abstract:**

Connexin-containing gap junctions mediate the direct exchange of small molecules between cells, thus promoting cell–cell communication. Connexins (Cxs) have been widely studied as key tumor-suppressors. However, certain Cx subtypes, such as Cx43 and Cx26, are overexpressed in metastatic tumor lesions. Cyclic adenosine monophosphate (cAMP) signaling regulates Cx expression and function via transcriptional control and phosphorylation. cAMP also passes through gap junction channels between adjacent cells, regulating cell cycle progression, particularly in cancer cell populations. Low levels of cAMP are sufficient to activate key effectors. The present review evaluates the mechanisms underlying Cx regulation by cAMP signaling and the role of gap junctions in cancer progression and metastasis. A deeper understanding of these processes might facilitate the development of novel anticancer drugs.

## 1. Introduction

Cyclic adenosine monophosphate (cAMP) is a small molecule that acts as a second messenger in mediating intracellular signal transduction [1,2]. Early studies indicated that cAMP signaling depends primarily on its activation of protein kinase A (PKA) [3]. However, four other key mammalian effector protein families have also been shown to be direct targets of cAMP, including the exchange protein activated by cAMP (EPACs1 and 2), the cyclic nucleotide gate channels, proteins containing Popeye domains, and a cyclic nucleotide receptor involved in sperm function [4]. Notably, cancer research has primarily focused on PKA and EPAC [5].

The cAMP/PKA signaling pathway is involved in the invasion, migration, adhesion, clonal growth, and other malignant phenotypes of cancer cells, including glioblastoma, ovarian cancer, colorectal cancer, breast cancer, and also pituitary tumors [6,7,8,9,10]. In particular, PKA phosphorylation of vasodilator-stimulated phosphoprotein (VASP) enhances the invasion and metastasis of esophageal squamous cells [11]. In addition, a related study on CIP4 PKA phosphorylation showed its regulation of cancer metastasis [12]. Moreover, TGFβ/PKA was found to be reportedly involved in colon cancer metastasis [13]. Therefore, PKA appears to be pivotal for malignant transformation.

Research on cAMP signaling in the cancer field has primarily aimed at identifying effective therapeutic targets for cancer [2,14]. However, there are several important studies focusing on the cross-talk among cAMP, Cx, and gap junction intercellular communication (GJIC). Connexins (Cxs), with 21 subtypes in humans, form both gap junctions and hemichannels that mediate the communications between two adjacent cells and between intracellular, and extracellular microenvironments, respectively [15,16,17,18]. Cxs are regulated by the cAMP signaling pathway, whereas Cx channels mediate the transfer of cAMP between cells via GJIC and in some circumstances, release to the extracellular milieu via Cx hemichannels. The significance of this phenomenon in cancer biology has been recently highlighted by several independent studies. Research conducted previously primarily focused on the mechanisms underlying the tumor-suppressor function of Cxs within a single cell, but more recently, attention has shifted toward an understanding of role of Cx channels in cAMP signaling in relation to the entire cell population [19,20]. One recent study demonstrated that cAMP exchange between cells via gap junctions limits cell cycle progression [21], proposing a potentially unifying model that could explain how gap junctions modulate tumor progression. Hepatic epithelial cells are instead very sensitive to the levels of both cAMP and Cxs. For example, increased functionality of the gap junctions or elevated levels of cAMP have been shown to reduce the tumorigenicity of HCC cells [22]. However, these mechanisms, which are pivotal to tissue homeostasis, require further investigation.

Cxs are direct targets of cAMP/PKA signaling [23], with the assembly of Cx43 channel clusters between cells being modified by cAMP signaling [24]. The cAMP/PKA pathway regulates the expression and phosphorylation of Cxs. Persistent hyperglycemia reduces *Cx36* mRNA levels in insulin-secreting cells in a rat model of continuous glucose infusion, and blocking the cAMP/PKA pathway attenuates this effect [25]. The cAMP/PKA pathway phosphorylates Cxs, and like in the zebrafish retina, Cx35/36 is directly phosphorylated by PKA at serine 110 and 276/293 to regulate photoreceptor coupling [26]. PKA directly phosphorylates Cx43 in rats, forming a Cx43 gap junction regulated by follicle-stimulating hormone [27], whereas the PKA–ezrin–Cx43 axis directly regulates GJIC in the human placenta to trigger trophoblast cell fusion [28]. Therefore, whereas Cx channels mediate the intercellular transfer of cAMP, they are also regulated by it.

## 2. Regulation of Cx Expression by cAMP/PKA Signaling in Cancer Cell Growth and Primary Cancer Progression

Cx expression has long been correlated with the occurrence, progression, and metastasis of cancer. Sixty years ago, Loewenstein and colleagues observed the loss of intercellular coupling in numerous cancers, and Cxs have been characterized as tumor suppressors in several cancer subtypes [19,29]. The overexpression of Cxs has been shown to inhibit primary tumor growth and progression in pancreatic cancer cells and breast cancer [19], as is the case for Cx43 in colorectal cancer cells [30]. However, recent studies have shown that Cx expression is tissue- and stage-specific in cancer. For example, the expression profile of Cx43 in breast cancer is largely dependent on the intrinsic subtype of breast cancer, but the majority of breast cancer cells exhibit reduced Cx43 expression or its aberrant localization in the cytoplasm [31]. In addition to those in the primary tumor, the changes in Cx expression are directly correlated with cancer metastasis. The metastasis of tumor cells from a primary site to distant foci is perhaps the most critical stage in cancer progression, as this is what typically leads to terminal outcomes for patients. In this phase, the role of Cxs is typically opposite, as the upregulation of Cx expression is most often positively associated with metastatic cancer, such as the role of Cx43/Cx26/Cx30 in metastatic breast cancer and prostate cancer bone metastasis [32,33,34,35,36]. Furthermore, Cxs are involved in entry (intravasation) and exit (extravasation) from the bloodstream [32,37,38], and several Cxs, including Cx43/Cx26/Cx30, have been implicated in the invasion of remote tissues, like prostate cancer bone metastasis (Cx43), breast cancer lymphatic vessel invasion (Cx26), and breast cancer and melanoma brain metastasis (Cx43 and Cx26) [32,33,34].

cAMP signaling plays a role in cancer cell migration; however, it does not alter Cx43 and Cx32 expression, but rather upregulates Cx26 [39]. In addition to Cx expression, it is noteworthy that cell movement depends on the formation of gap junction channels [40], especially the channels formed by Cx26 [41]. This might partially explain the response of Cx26 expression to cAMP signaling.

In several systems, cAMP via PKA appears to be critical in regulating Cx expression, although whether it exerts positive or negative effects appears to depend on the tissues and tissue-specific Cx expression profile. However, these studies were not conducted on cancer cells. For example, continuous hyperglycemia results in a decline in the mRNA levels of rat *Cx36* (*rCx36*) in insulin-secreting cells in rats administered a continuous glucose infusion, an effect that is blocked by the inhibition of cAMP/PKA signaling [25]. In contrast, prostaglandins have been shown to induce the expression of rat Cx43 (rCx43), which can be reversed by the adenylate cyclase inhibitor SQ22536 or the PKA inhibitor H89 in mesangial and bladder smooth muscle cells [42].

**Figure 1:**
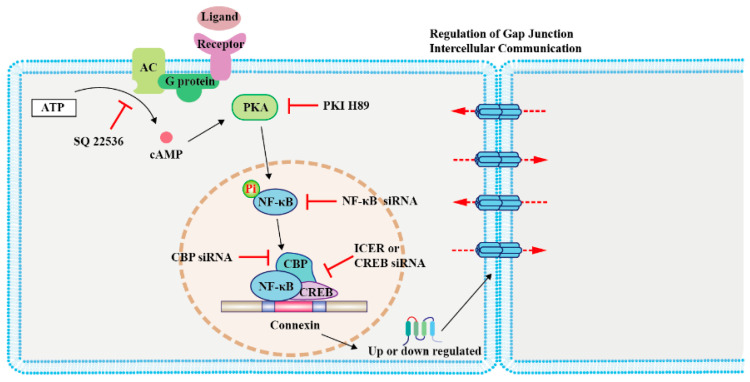
Regulation of connexin (Cx) expression by cAMP/PKA signaling in modulating cancer cell growth and primary cancer progression. The schematic illustrates the mechanism involved in the cAMP signaling-mediated regulation of Cx levels. Activation of the G-protein-coupled receptor on the cell membrane by ligands leads to cAMP synthesis by AC. cAMP diffuses into the cell and activates its effector protein PKA, which phosphorylates NF-κB. Subsequently, NF-κB, CBP, and CREB form a complex to regulate the transcription of Cxs [25,42,43,44]. Abbreviations: AC, adenylyl cyclase; PKA, protein kinase A; NF-κB, nuclear factor kappa-B; CBP, CREB-binding protein; CREB, cAMP response element-binding protein; PKI, PKA inhibitor; and ICER, inducible cAMP early repressor I.

The cAMP response element-binding protein (CREB) can directly regulate the transcription of *Cx43* by forming complexes with nuclear factor kappa-B (NF-κB) and CREB-binding protein (CBP), the latter two being important effectors of cAMP [43]. The modulation of CREB enhances rCx43 expression [43]. Conversely, inducible cAMP early repressor I decreases rCx36 expression in pancreatic islets in rats [25]. Moreover, oxytocin regulates mCx43 expression by activating cAMP/PKA signaling and enhancing the expression of nuclear factor kappa-light-chain enhancer in mouse embryonic stem cells [44] (Figure 1). Although these studies have been done with non-cancer cells, a similar mechanism could be adopted by certain types of cancer cells. Moreover, the NF-kB/CBP/CREB complex can regulate the expression of target genes both positively and negatively [45]; thus, studies that address both of these circumstances should be considered. It would be interesting to explore the effect of the cAMP/NF-κB/Cx axis in cancer apoptosis.

## 3. cAMP/PKA Signaling Mediates the Phosphorylation of Cxs to Promote Cancer Cell Migration and Malignant Transformation

The functional regulation of GJIC, especially Cx phosphorylation, is necessary for cancer cell migration [46]. The phosphorylation of rCx43 at S255, S279, and S282 has been reported, which either opens or closes the gap junction [47,48,49]. Cx43 contains multiple phosphorylation sites regulated by kinase-mediated signaling pathways that interact with other proteins, including PKA [49,50]. A previous study indicated that stimulating the cAMP pathway regulates hemichannel switching, as well as endocrine and paracrine signaling [51]. Another study showed that dipyridamole induces cAMP production to post-translationally modify bovine Cx43 (bCx43), resulting in increased gap junction coupling of endothelial cells [52].

**Figure 2:**
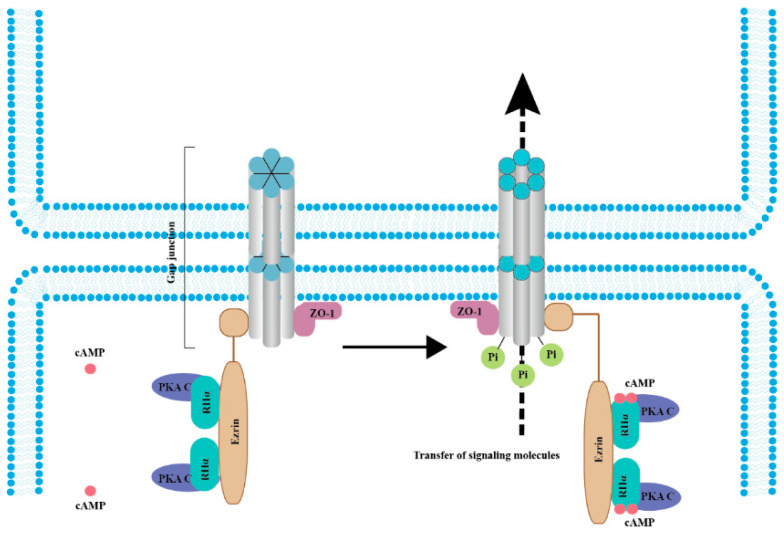
cAMP/PKA signaling mediates the phosphorylation of connexins (Cxs) to promote cancer cell migration and malignant transformation. In the resting state (left panel), the whole complex consists of Cx, ZO-1, Ezrin, and a PKA pool anchored by Cx-bound ezrin. When cAMP levels increase (right panel), PKA regulatory subunits bind to four cAMP molecules, resulting in activated PKA catalytic subunits, increased Cx phosphorylation, and gap junction-mediated intercellular coupling [26,53,54,55]. Abbreviations: RIIα, PKA regulatory subunits and PKA C, PKA catalytic subunits.

Interestingly, Cx43 phosphorylation by PKA seemingly does not directly affect the channel but has been linked to trafficking. rCx43 phosphorylation can be influenced by PKA localization and by other factors. For example, the tight junction protein 1 (ZO-1) binds both PKA and Cx43, bringing the kinase in proximity to its targets on Cx43, which leads to gap junction channel opening [53]. In fact, separation from ZO-1 is necessary for gap junction closure [53]. It was also determined that the complex formed by ezrin, rCx43, and ZO-1 brings PKA in proximity and phosphorylates rCx43, promoting the opening of the gap junction channels (Figure 2) [54]. In contrast, PKA inhibition diminishes rCx43 phosphorylation [55]. These data indicated that the cAMP/PKA pathway is instrumental in the phosphorylation of rCx43.

The accelerated proliferation of cancer cells is reportedly associated with Cx43 phosphorylation. Phosphorylated Cx43 is upregulated during hyperplasia, atypical hyperplasia, fibroadenoma, and especially in invasive cancer [56]. Similarly, human gliomas exhibit the significant overexpression of phosphorylated Cx43 [57]. Together, these data indicate that the malignant proliferation of cancer cells is related to the phosphorylation of Cx43. However, in many of these studies, the particular kinases mediating the phosphorylation reaction have not been identified. Notably, PKC, extracellular regulated protein kinases, and tyrosine kinases like c-src can phosphorylate Cx43, which generally causes the closure of the channels [58]. In non-cancer, specifically in the nervous system, the phosphorylation of goldfish Cx35 (gCx35) is associated with the dynamic control of gap junction coupling in the zebrafish Mauthner neuron [59]. Furthermore, this has been observed in the zebrafish retina, where cAMP activates the channels and mediates the activity-dependent potentiation of coupling; PKA activity plays a key role in this process [26]. Therefore, cAMP-activated PKA phosphorylates Cx to regulate GJIC. Cx35/36 phosphorylation by PKA seems to directly affect the probability of channel opening. However, caution is needed in interpreting these results, as the effects observed by blocking PKA do not necessarily imply direct phosphorylation of the Cx protein, unless this is actually demonstrated.

## 4. Gap Junction Channels Mediate Transfer of cAMP

In addition to considering Cx as targets of cAMP-mediated signaling, it is also relevant to consider the roles of Cxs and GJIC in its propagation. However, it is crucial to consider that not all Cxs form channels with similar permeability for cAMP. For example, cAMP permeability is much higher through rCx43 than Cx50 channels expressed in HeLa cells [60]. Furthermore, rCx43, mCx46, and Cx50 present a different permeability for cAMP in lens cells [60]. In a more comprehensive study using a more indirect reporter of cAMP transfer, the order of cAMP permeabilities through different Cxs is as follows: Cx43 > Cx26 > Cx45 = Cx32 > Cx47 > Cx36 [61]. However, direct measurements of cAMP transfer by Cxs expressed in oocytes show that Cx26 > Cx43 ~ Cx32 (Nicholson, personal communication). For example, cAMP transfer between cells mediated by Cx43 modifies the expression of key effector molecules and genes in interconnected osteosarcoma cells [62].

## 5. Cxs and GJIC Regulate the Cell Cycle and Limit the Rate of Mitosis of the Tumor Cell Population

**Figure 3:**
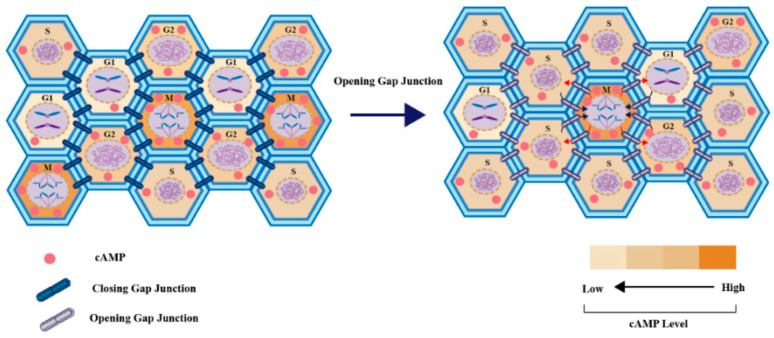
Gap junction-mediated transfer of cAMP limits the rate of mitosis of the whole tumor cell population. Changes in cAMP levels alter PKA activity to regulate cell cycle progression. Low levels of PKA activity help cells progress through the G1 and S phases to interphase. However, high levels of PKA activity are needed for cells to enter and exit the mitotic cycle. In the absence of gap junction-mediated communication, different cAMP levels in each cell result in different cell cycle stages in the population. However, the cell groups that maintain communication throughout the cell cycle exhibit a uniform distribution of cAMP, thereby, resulting in the dilution of cAMP (and decrease in P-PKA) in M-phase cells (leading to the inhibition of mitotic processes) and an increase in cAMP and PKA activity in G1/S-phase cells (leading to partial G1 phase arrest). Thus, cAMP redistribution delays the M phase and cell cycle progression through the interphase [21,63].

Studies have shown that Cx43 is an important regulator of tumor cell proliferation via the cAMP pathway [63] and a necessary factor for effective cell cycle progression [21] (Figure 3). The upregulation of Cx43 and cell cycle inhibitors p21cip1, p27kip, and p19ink4 leading to the cessation of proliferation indicates that Cx43 might be associated with cell cycle regulation in Sertoli cells [64]. In addition, Cx43 is related to phosphodiesterase 4 (PDE4), cAMP (Epac1), and cyclin E1 and synergistically promotes rectal cancer [65].

cAMP-activated PKA is likely central to impacts on cell functions [66]. Whereas PKA activation depends on the level of cAMP [67], it might not always be reflected only by global cAMP levels or its localization between individual cells (cAMP regionalization) or even within varying regions of a single cell [66]. For example, the activation levels of PKA in HeLa cells in the presence or absence of gap junctions were shown to not correlate with the global levels of cAMP in the entire cell population, but rather, reflect the cAMP distribution in individual cells of the whole cell population. cAMP has long been known to oscillate during the cell cycle, with higher levels promoting exit from mitosis (G2-M phase) and lower levels required for DNA replication (G1 phase). Comparing tumor cell lines expressing different Cxs, Chandrasekhar et al. showed that Cx26 specifically redistributes cAMP to all cells, eliminating the differences in cAMP levels at various mitotic stages, creating a general suppression of proliferation [21]. Interestingly, the effects were specific to gCx26, as other Cxs, such as Cx43, are closed via phosphorylation during the G2–M phases. These studies implied that cAMP transport between tumor cells via open gap junctions limits the rate of mitosis of the whole tumor cell population, potentially explaining why more rapidly growing tumors frequently reduce intercellular coupling. However, the complete mechanisms underlying the regulation of cancer tissue homeostasis by cAMP remain to be elucidated.

The cAMP redistribution model of gCx26 [21] is linked to cell cycle involvement in adhesion, migration, and metastasis; this model explains how Cx channels work by regulating different underlying mechanisms of cancer progression. The goldfish Cx26 channel transmits cAMP, thereby mediating cell cycle progression [21]. Cx43-mediated cell adhesion might be involved in activation of the cell cycle via adhesion molecules, like ZO-1 [68]. Moreover, the biological effect of G protein-induced activation of cAMP signaling on cancer cell proliferation indicates that cell adhesion could be linked to cell proliferation. G protein-coupled receptors are highly associated with cell adhesion; thus, cancer cells stimulate cell proliferation possibly via their adhesive properties [69,70]. G protein-coupled receptor 64 (GPR64) is overexpressed in invasive SG adenoma [71]. GPR64 knockout reduces cAMP levels and the phosphorylation of CREB (decreasing the p-CREB/CREB ratio), resulting in arrest of the GH3 tumor cell cycle [71]. Similarly, eicosapentaenoic acid was found to repress the ovarian cancer cell line ES2 via GPR30 and inhibit the growth of ovarian clear-cell carcinoma [72].

## 6. Role of Cx–cAMP in Cancer Metastasis

There are limited studies connecting Cxs with cAMP signaling in cancer metastasis. PKA was shown to be involved in regulating cancer metastasis [12]. Furthermore, GPCR is an upstream regulator of cAMP, along with downstream cAMP effectors such as PKA [73]. Although numerous studies support the role played by Cx and cAMP in cell cycle progression, further experiments are warranted to determine if Cx-cAMP is associated with cancer metastasis.

## 7. Potential Strategies Targeting cAMP and Cx for the Bystander Effect

The modulation of gap junction function can improve cancer tissue sensitivity to chemotherapy and is a potential target for cancer treatment [2]. Effective cancer treatment usually requires a combination of chemotherapeutic regimens since cancer tissues often show resistance to a single drug. Over the years, various categories of anticancer drugs have been discovered, such as natural compounds [2] and peptides [74], which effectively inhibit cancer progression. However, the combinatorial use of these drugs to overcome cancer multidrug resistance is key to achieving efficacious clinical treatment. Studies pointed out that the activation of PKA was sufficient to induce resistance to tamoxifen in breast cancer [75]. Mutations in adenylate cyclase (ADCY1), which catalyzes ATP to cAMP, impact drug efficiencies in various cancers, such as lung cancer, esophageal cancer, and colorectal cancer [76]. It is thus reasonable to explore a correlation among ADCY1, Cx, and cAMP.

Prior studies have provided evidence of employing cAMP in cancer treatment for the modulation of GJIC levels. A combination of retinoic acid (RA), which regulates cell proliferation and differentiation, and cAMP improves hepatocellular carcinoma cell response to RA treatment; this treatment augments E-cadherin, Cx26, and Cx32 expression [23]. Moreover, dibutyryl cyclic (db)-cAMP enhances the neighboring bystander effect by improving medulloblastoma chemosensitivity through a pathway mediated by Cx43 and the apoptosis blocker BCL2 [77]. Conversely, a study involving the application of photodynamic therapy (PDT) to malignant tumors indicates that the inhibition of Cx32/Cx26-mediated GJIC enhances the sensitivity of malignant cells to PDT [78]. However, this is a contentious topic in the field, requiring additional evidence to bolster this notion. GJIC remains crucial for cancer drug sensitivity, and targeting cAMP signaling is an important method to improve GJIC.

Notably, GJIC comprises a concerted regulatory system for cAMP sensitivity among tissues and cells [79]. This system can inhibit cancer tissue progression by opening the GJIC in cancer cells and healthy tissues, allowing cAMP to enter cancer tissues and activate tumor suppressor-associated pathways. The physiological effect of drug-induced increased synthesis of endogenous cAMP on the growth arrest of co-cultured murine fibroblasts provides a feasible reference for this hypothesis [75]. Therefore, this regulatory system for cAMP sensitivity might serve as a novel therapeutic strategy for cancer. However, the caveat of enhancing GJIC in cancer treatment is that enhanced GJIC is also associated with increased metastasis. Therefore, prior to implementing such combinatorial approaches, we should first understand the specific mechanisms so we can mitigate primary tumor growth without promoting a metastatic phenotype.

## 8. Conclusions and Future Perspectives

Cx, a marker of cancer progression, plays a major role in the early etiology (associated with reduced Cx function) and later metastasis (associated with enhanced Cx function) of cancer. cAMP flows between cancer cells through Cx channels, which can affect cancer proliferation and chemoresistance by disseminating drugs through the tumor. In turn, Cx channels are themselves regulated by cAMP and its effector, PKA. Therefore, detailed, future studies are warranted on the cross-talk between cAMP and Cx to elucidate their complex interaction in cancer growth and metastasis and provide new insights into the development of therapeutics for difficult-to-treat malignant cancers. These mechanistic understandings will be critical, given that Cxs appear to play a multifaceted role, inhibiting the growth of primary tumors and facilitating drug sensitivity while simultaneously enhancing the metastatic potential. Moreover, it would be interesting to determine the effect of increased cAMP or Cxs on proliferation in a physiological and tumoral system and potential difference in cell proliferation in normal and cancer cells ought to be carefully considered. The potential scientific questions regarding cAMP/Cxs in cancers, including that the difference in different cancer types, types of connexins, in primary and advanced tumor and metastasis, need to be clarified in the future.

In additional to cAMP/PKA in regulating Cxs, it worth to note that other signaling pahtways are reported to be involved in the regulation of Cxs, including Wnt, RAS-RAF-MAPK pathway [80,81]. Wnt1-expressing clones in the rat neural-crest-derived cell line PC12 display an increased electrical and chemical coupling, which coincides with an increased expression of Cx43 mRNA and protein, whereas other connexins, Cx26, Cx32, Cx37, Cx40, and Cx45, are not upregulated. They also showed Wnt1 and Li+, an ion that mimics Wnt signaling, increased transcription of rat Cx43 potentially via TCF/LEF binding elements. Moreover, neonatal rat cardiomyocytes responded to Li+ by accumulating the effector protein β-catenin and by inducing Cx43 mRNA and protein markedly, and that by transfecting a Cx43 promoter P1-reporter gene construct into cardiomyocytes, the inductive effect of Wnt signaling was transcriptionally mediated [80]. Besides Wnt signaling, Carystinos et al. found that both Cx43 mRNA and protein levels are increased in H-Ras-overexpressing NIH3T3 cells and that the cells also have enhanced Cx43 promoter P2 activation, which is inhibited by the MEK1 inhibitor. Furthermore, they also mapped a minimal DNA sequence essential for the Ras-mediated Cx43 upregulation and that a protein complex in nuclear extracts from NIH3T3-Ras and MCF7-Ras selectively recognizes a consensus sequence, AGTTCAATCA, located at positions +149 to +158 of the Cx43 promoter P2, in which complex the 90-kDa heat shock protein (HSP90) and c-Myc are identified as constituents [81].

## Data Availability

The data presented in this study are available on request from the corresponding author.
